# A case of atrophic dermatofibroma: a possible role of matrix metalloproteinase-2^☆^

**DOI:** 10.1016/j.abd.2022.06.011

**Published:** 2023-11-27

**Authors:** Misaki Kusano, Toshiyuki Yamamoto

**Affiliations:** Department of Dermatology, Fukushima Medical University, Fukushima, Japan

*Dear Editor,*

A 47-year-old male visited our department complaining of an asymptomatic nodule on the lower extremity, which appeared 5 years previously. He was otherwise healthy and did not recognize any triggering events such as insect bite or minor trauma. Physical examination showed a 5-mm-sized brownish dermal nodule with a central depression on the left thigh (Fig. 1A). Dermoscopy showed a central, reddish-colored area with scales surrounded by a brownish pigment network (Fig. 1B). The nodule was surgically removed with a 2-mm margin under local anesthesia. Histopathological examination showed a central depressed area and dermal atrophy (Fig. 2A). Higher magnification revealed the proliferation of fibroblastic tumor cells in the dermis and hyperplasia of the overlying epidermis (Fig. 2B). Factor XIIIa was positively stained, but CD34 was negatively stained. Elastica van Gieson stain revealed decreased elastic fibers in the dermis (Fig. 2C). Immunohistochemistry was performed using antibodies against Matrix Metalloproteinase-2 (MMP-2), MMP-7, MMP-9 and MMP-12, and intense expression of MMP-2 was observed in the fibroblastic tumor cells (Fig. 2D).

**Figure 1:**
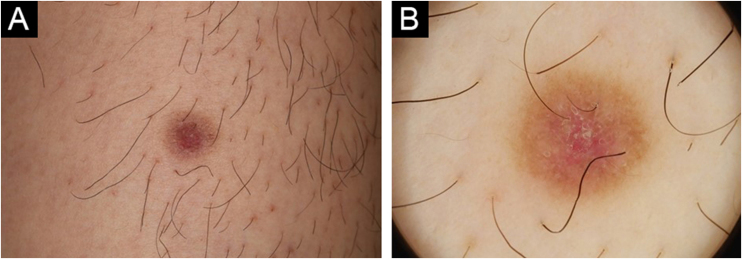
(A) Clinical appearance of the brownish nodule with a central depression. (B) Dermoscopy showing a central reddish patch with white-yellow scales surrounded by a brownish pigment network.

**Figure 2:**
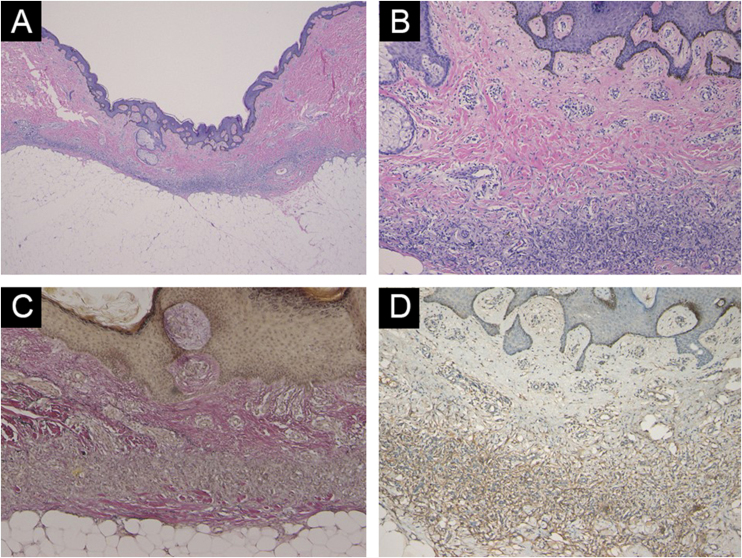
Light Microscopy -(A) Lesion with a central depression showing dermal atrophy (Hematoxylin & eosin, ×40). (B) Higher magnification showing proliferation of fibroblastic tumor cells in the dermis and hyperplasia of the overlying epidermis (Hematoxylin & eosin, ×200). (C) Decreased elastic fibers in the atrophic dermis (Elastica van Gieson, ×200). (D) Intense expression of MMP-2 in the fibroblastic tumor cells, magnification ×200.

Atrophic dermatofibroma is a rare form of dermatofibroma and is clinically characterized by a solitary brownish nodule or plaque with a central umbilication.^1^, ^2^ Its dermal thickness is usually half of the thickness of the adjacent dermal tissue. In a recent review of atrophic dermatofibroma in 64 patients, the most common locations were shoulder (25%), lower extremity (23.4%), and back (17.2%).^1^ Because of the characteristic clinical features such as brownish nodules or plaque with central depression, the clinical diagnosis is not so difficult. Dermoscopic examination shows a patchy pigment network multiple scar-like white patches, and pink-reddish coloration,^3^, ^4^ which may be of some help for the clinical diagnosis of atrophic dermatofibroma.

The pathomechanism of dermal atrophy in atrophic dermatofibroma is unknown. Previous studies revealed that elastic fibers are either decreased or absent in atrophic dermatofibromas. Recently, overexpression of MMP-1 by tumor cells has been reported in atrophic dermatofibroma.^5^ In addition, in the present study, we have found that MMP-2 was strongly expressed in the fibroblastic cells, whereas expression of other MMPs such as MMP-7, MMP-9, and MMP-12 was not observed. The limitation is that we did not examine MMPs expression in ordinary dermatofibromas without atrophy. Therefore, we cannot conclude that enhanced expression of MMP-2 is the main cause of such a characteristic feature of atrophic dermatofibromas. Nevertheless, MMP-1 and MMP-2 may play an important role in the degradation of connective tissues in atrophic dermatofibromas, and further studies are necessary.

## Financial support

None declared.

## Author’s contributions

Misaki Kusano: Data collection, analysis, and interpretation; Preparation and writing of the manuscript.

Toshiyuki Yamamoto: Manuscript critical review; Approval of the final version of the manuscript.

## Conflicts of interest

None declared.
